# Observational report: Improved outcomes of transscleral cyclophotocoagulation for glaucoma patients

**DOI:** 10.1097/MD.0000000000006946

**Published:** 2017-06-08

**Authors:** Rick Edward Bendel, Michael Ty Patterson

**Affiliations:** Mayo Clinic Foundation, Jacksonville, FL.

**Keywords:** cyclophotocoagulation, G-probe, glaucoma, micropulse cyclophotocoagulation, transscleral cyclophotocoagulation

## Abstract

The purpose of this article is to evaluate the improved safety and efficacy of transscleral cyclophotocoagulation (TSCPC) by performing it in the operating room.

This is a retrospective review of 17 eyes of 16 patients who received TSCPC for uncontrolled glaucoma on maximum tolerated medication.

Mean intraocular pressure (IOP) prior to surgery was 30.85 ± 6.24 mm Hg and was reduced to 14.48 ± 3.53 mm Hg after treatment for an average reduction in IOP of 48.56% at the final visit (*P* < .001). Visual acuity was measured at the final follow-up visit and was stable in 13 eyes (76.47%), improved in 2 eyes (11.75%), and decreased in 2 eyes (11.75%). Nine of the eyes (52.94%) saw a reduction in the number of medications taken, whereas 8 had no change. Two eyes had resolved complications of cystoid macular edema (CME) and subconjunctival hemorrhages. The overall success rate is determined to be 88%.

TSCPC performed in the operating room may have greater safety and efficacy for patients with uncontrolled glaucoma.

## 1 Introduction

The use of cyclophotocoagulation procedures for the treatment of glaucoma have increased significantly in the past decade.^[1]^ Transscleral cyclophotocoagulation (TSCPC) is a method that aims to reduce intraocular pressure (IOP) by ablating the ciliary body to reduce the production of aqueous humor, and thus lowering pressure. Both contact and noncontact methods have been explored; however, direct contact allows for conjunctival and scleral compression, which leads to a more direct transfer of energy.^[2]^ The transmitted energy from the laser causes destruction of both the ciliary epithelium and blood vessels, which leads to coagulative necrosis of the ciliary body. TSCPC has proved to be a successful surgical method to lower IOP^[3–5]^ while not increasing visual field loss.^[6]^ However, many ophthalmologists hesitate to perform this procedure due to its painful nature and high risk of complications such as hypotony and phthisis bulbi.^[7,8]^ Trabeculectomy has often been considered the most effective method in controlling glaucoma, but the number of trabeculectomies performed worldwide is steadily decreasing.^[1]^ This trend is mainly due to the long-term morbidity associated with the procedure.^[9,10]^ Thus, glaucoma research is looking for alternatives with equal efficacy and far less complications. TSCPC presents to be a viable alternative if pain and complications are minimized.

In order to combat these issues, Iridex recently developed a new method of laser delivery known as MicroPulse Laser Therapy. This delivery mode uses repetitive, short pulses of laser administration separated by rest periods. It has been shown that this method allows for a more selective targeting of the ciliary body^[11]^; however, many physicians are noticing that the IOP-lowering ability may be compromised. Since the inception of this MicroPulse technology, many physicians have avoided performing the traditional continuous-wave TSCPC because of the perceived equal efficacy and decreased morbidity rate associated with the MicroPulse method. In our practice, we have continued to perform the continuous-wave TSCPC, but have opted to administer this procedure in the operating room (OR) rather than in the clinic. When performed in the OR, this procedure can be administered with heavy sedation, which has allowed for better tolerability for the patient enabling the surgeon to more accurately apply laser pulses.

In this case series, we evaluate the efficacy and safety of TSCPC in treating glaucoma when moved to the OR as opposed to being performed in the clinic. We also explore the possible advantages of using the continuous-wave method over the MicroPulse delivery system.

## 2 Patients and methods

A retrospective chart review was done on 17 eyes of 16 patients who received TSCPC for treatment of the following forms of glaucoma: pseudoexfoliation, primary open angle, angle recession, congenital, neovascular, traumatic, and other secondary forms of glaucoma (Table 1). Of these eyes, 12 (70.6%) had prior glaucoma procedures (Table 2). All operations were performed at the Mayo Clinic of Jacksonville, FL by a single surgeon between December 2011 and April 2016 in the OR. Prior to surgery, patients underwent a baseline examination, which included collection of best-corrected visual acuity (BCVA), IOP measured using Goldman Applanation Tonometry, demographic data, and ocular history. The inclusion criteria were presence of uncontrolled glaucoma on maximum tolerated glaucoma medications and an average preoperative IOP of >20 mm Hg. Success was defined as a postoperative IOP of 5 to 22 mm Hg while not increasing the number of glaucoma medications and no reduction of visual acuity.

**Table 1:**
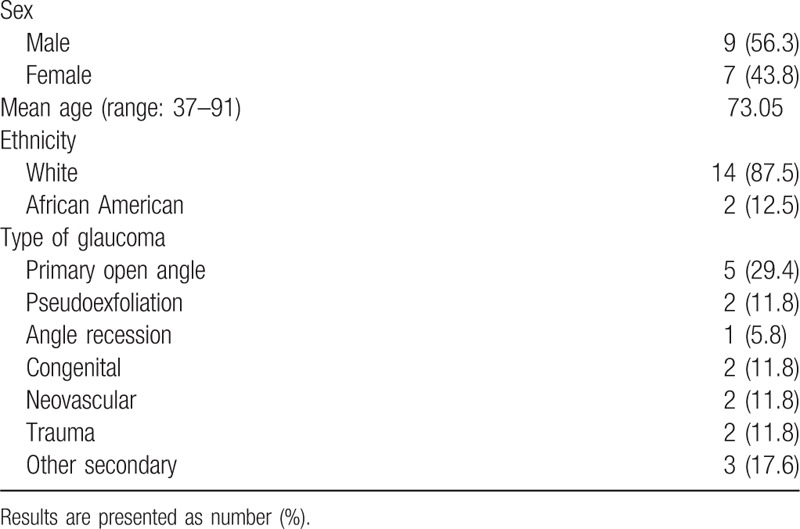
Demographic data for 17 eyes.

**Table 2:**
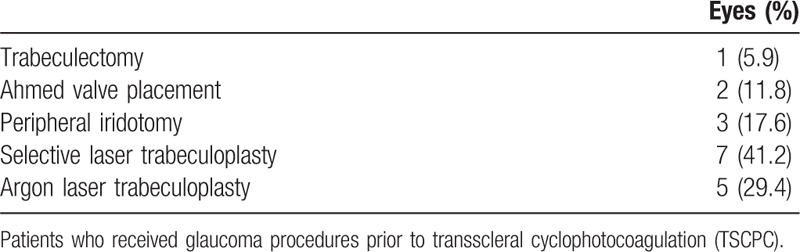
Previous glaucoma procedures.

The procedure was performed under monitored anesthesia care with topical lidocaine. An OcuLight SLx semiconductor diode 810 nm laser was used with the contact G-probe and applied by a single operator. Laser treatment consisted of 18 to 21 applications over 270° using an average of 2000 mW of power for 1 to 2 seconds. Prednisolone acetate 1% was given postoperatively for 1 week. The mean follow-up time was 156.3 ± 10.2 days (range: 3–50 months) with an average of 6.8 ± 2.2 visits. At each follow-up visit an examination was done that included IOP and BCVA measurements, an account of the number of glaucoma medications, a pain determination using a verbal analog scale in which the patient was asked to gauge their pain as no pain, mild pain, moderate pain, or severe pain and an examination for possible complications caused by the procedure. Pain was considered prolonged if the patient reported any discomfort for at least 2 consecutive visits. Statistical analysis of the difference in IOP and number of glaucoma medications before and after surgery was carried out using a Student *t* test in which a *P* value of <.05 was considered statistically significant. A Kaplan-Meier analysis was generated to determine survival rates using the previously described success definition to compensate for the varying follow-up times. All statistical analyses were performed on Microsoft Excel. This observational study was done with the approval of the Institutional Review Board.

## 3 Results

**Figure 1:**
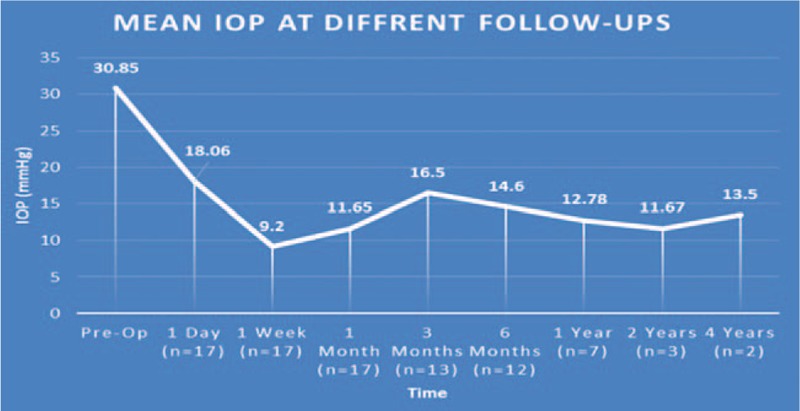
Intraocular pressures at different follow-up visits. IOP = intraocular pressure.

**Figure 2:**
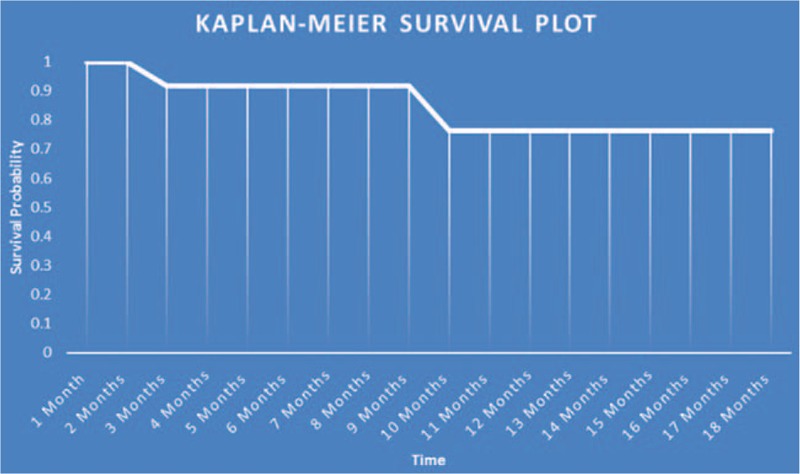
Kaplan-Meier survival plot.

A total of 17 eyes were enrolled in this study that had been previously diagnosed with uncontrolled glaucoma. Mean IOP prior to surgery was 30.85 ± 6.24 mm Hg and was reduced to 14.48 ± 3.53 mm Hg after treatment at each patient's final visit (follow-up range: 2–50 months). The average reduction in IOP was 48.56% at the final visit. Figure 1 compares preoperative IOP to postoperative measurements at various follow-up times. A Student *t* test was used to compare average preoperative IOP to each follow-up time and each interval gave a statistically significant reduction of IOP (*P* < .001). Visual acuity was measured at the final follow-up visit and was stable in 13 eyes (76.47%), improved in 2 eyes (11.75%), and decreased in 2 eyes (11.75%). However, 1 patient who had a decreased acuity suffered a visual cortex stroke during the follow-up period so the true number of eyes that experienced decreased vision attributable to the TSCPC procedure was considered to be 1 (5.88%) (Table 3). The number of glaucoma medications was recorded at the final visit and compared to the amount of preoperative medications. Of the 17 eyes, 9 (52.94%) saw a reduction in the number of medications taken while the rest saw no change (Table 4). A Student *t* test was used to assess the statistical significance of these findings and a *P* value of <.01 was determined to be significant. No major complications (hypotony, phthisis bulbi) were suffered; minor complications were seen in 2 (11.11%) of the eyes (Table 5). One of the patients saw both subconjunctival hemorrhage and cystoid macular edema (CME). With extended steroid and nonsteroidal drop regimens the CME fully resolved and did not recur with tapering. No prolonged postoperative pain was reported. Given the previously discussed success parameters, an overall success rate of 88.24% was determined. The Kaplan–Meier survival analysis (Fig. 2) shows a stable survival rate approximately 80% up to 18 months postop.

**Table 3:**
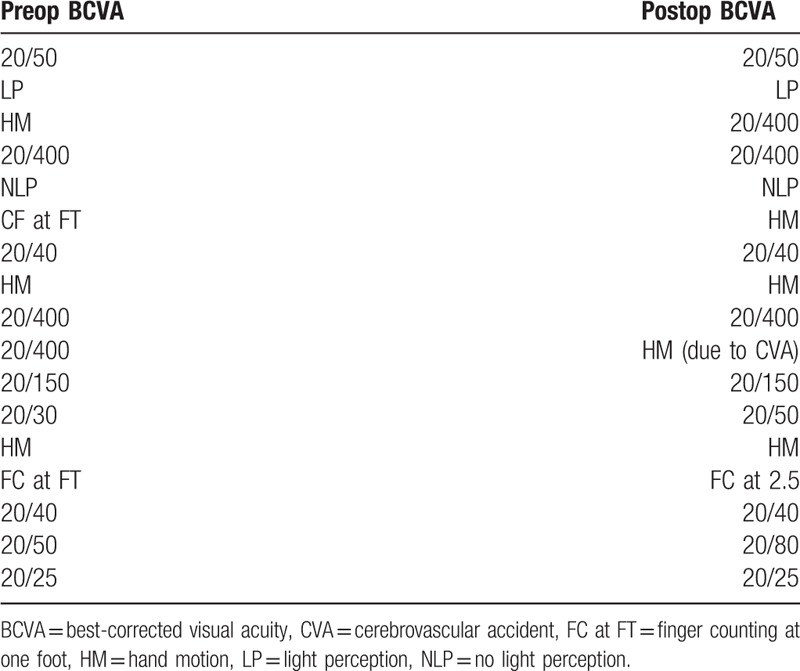
Visual acuity before and after transscleral cyclophotocoagulation.

**Table 4:**
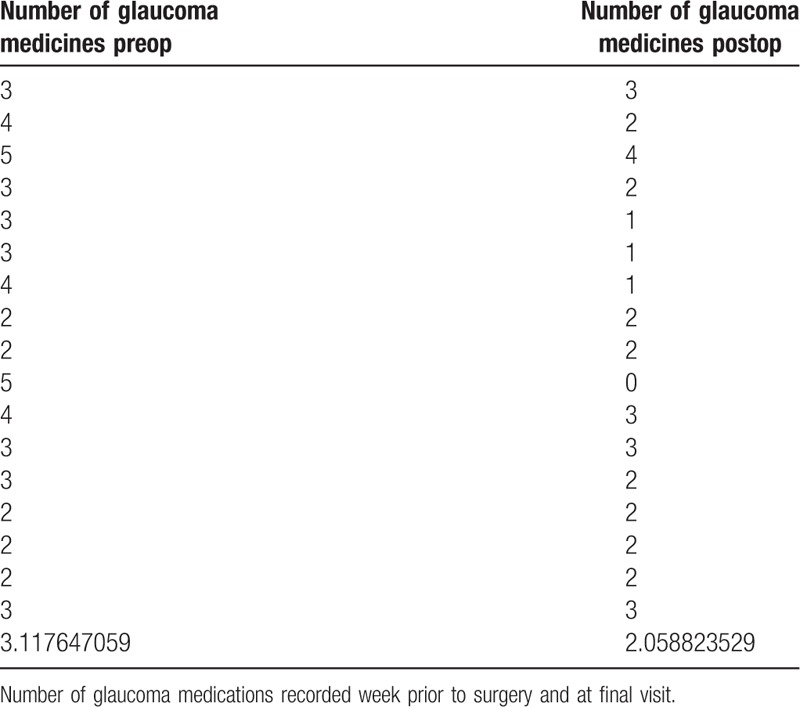
Number of antiglaucoma medications before and after transscleral cyclophotocoagulation.

**Table 5:**
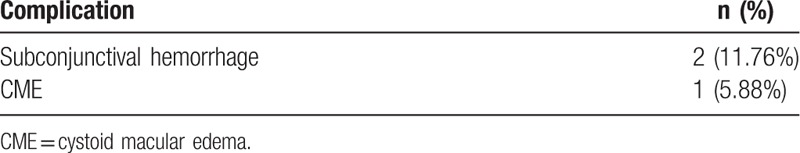
Complications after transscleral cyclophotocoagulation.

## 4 Discussion

In recent years TSCPC using the G-probe has become a more viable tool to treat refractory glaucoma; however, many surgeons still hesitate to use this procedure due to the potential for serious complications and its painful nature. Many studies confirm the IOP-lowering ability of TSCPC^[3–5]^; however, there is conflicting data on the complication rates of this procedure. Ramli et al^[12]^ and Murphy et al^[13]^ both reported that there was a significant risk of developing serious complications such as hypotony, while Osman et al^[14]^ and Ansari and Gandhewar^[15]^ reported no significant risk for development of these complications. This conflicting data, along with many reports of operative and postoperative pain associated with this procedure^[16,17]^ merit consideration on how to maintain good outcomes while lowering complications and pain. Our series saw a significant decrease in IOP (*P* < .0001) in all 17 eyes at their final visits, and all patients had a final IOP in the successful range (5–22 mm Hg) at the final follow-up. In all cases, the number of glaucoma medications was either maintained or reduced. Another important variable considered in this study was intraoperative or prolonged pain, which was reported in 0 of the 17 eyes. The 2 patients who were considered failures both underwent a second TSCPC procedure due to uncontrollable IOP and progressing vision loss. Of these 2 patients, one of them received a successful outcome after only 1 additional application, whereas the other could not maintain a stable IOP and experienced substantial visual field loss after multiple applications of the G-probe. It is useful to point out that this case was rather complex and the patient received TSCPC after multiple failed procedures. Given this, a success rate of 88.24% is rather conservative and is probably higher. To our knowledge, no previous study on TSCPC reports a success rate this high with a complication and pain percentage this low after only 1 treatment. Prior to this study, our clinic performed all TSCPC procedures in the clinic and saw a success rate of 33%. After moving TSCPC to the OR, we saw an increase in success rates which points to the possibility of a correlation between improved outcomes and OR surgical practices. Osman et al^[14]^ reported a similar reduction in IOP in a similar cohort of patients when performed in the clinic. However, they also saw a significantly higher rate of pain and complications.

There are several reasons we believe why performing TSCPC in the OR could produce more successful outcomes. First, when performed in an OR the patient can receive much better pain control by being placed under monitored anesthesia which would allow for much better tolerability during the procedure and thus more accurate laser applications. The G-probe by Iridex is sold as a single use probe and is priced anywhere from 100 to 220 US dollars. Many surgeons reuse the probe up to 50 times to cut down on surgeon's fees. Repeat use of the G-probe leads to both a fluctuation in energy output^[18]^ and a possibility of contamination^[19]^. However, in our practice when TSCPC is performed in the OR, the cost of the G-probe is covered by the OR facility fee. This could reduce the financial burden for surgeons in a similar situation and make them more likely to use the G-probe as directed, which also may allow for greater efficacy and better safety.

In comparing our outcomes to those of the MicroPulse delivery system, we found a similar cohort of patients who underwent MicroPulse TSCPC. Kuchar et al^[20]^ reported an average reduction in IOP of 40%, a success rate of 73.3% after initial treatment in 19 eyes using a similar definition of success, and a complication rate of 5.3%. Comparing our outcomes to this study, we see that the continuous-wave method of TSCPC has a stronger IOP-lowering ability and even though we see a greater complication rate in our cohort, we believe that due to the small number of patients in each study and the mildness of our complications that the complication difference between the groups is not significant. We feel that a greater number of patients are required to draw any true conclusions about the difference in complication rates of the 2 delivery methods. However, MicroPulse TSCPC lowers pressure in a much more passive manner by using short bursts of energy to increase uveoscleral outflow while continuous-wave TSCPC is more destructive to the ciliary body. This, along with the fact that the MicroPulse technology allows for no scaring, provides obvious safety advantages over conventional TSCPC. We believe that electing to perform either MicroPulse or conventional TSCPC should be based on the target postoperative IOP and all factors, including possible safety advantages, should be taken into account.

## 5 Conclusions

Our study suggests that TSCPC, when performed under heavy sedation and proper G-probe use is employed, leads to better outcomes and a greater clinical efficacy. We conclude that TSCPC should still be considered earlier for treating refractory glaucoma and can be used in a variety of glaucoma types.
